# Study of dosimetric properties of flattened and unflattened megavoltage x ray beam on high Z implant materials

**DOI:** 10.1002/acm2.12451

**Published:** 2018-09-28

**Authors:** Tamilarasan Rajamanickam, Sivakumar Muthu, Perumal Murugan, Muddappa Pathokonda, Krishnamoorthy Senthilnathan, Narayanasamy Arunai Nambi Raj, Padmanabhan Ramesh Babu

**Affiliations:** ^1^ Department of Radiotherapy Sri Shankara Cancer Hospital & Research Centre Bengaluru Karnataka India; ^2^ Department of Physics SAS Vellore Institute of Technology Vellore Tamil Nadu India; ^3^ Centre for Biomaterials, Cellular and Molecular Theranostics Vellore Institute of Technology Vellore Tamil Nadu India

**Keywords:** back scatter dose perturbation factor, flattening filter (FF), forward dose perturbation factor, mass attenuation coefficient, Monte Carlo, photon spectrum, unflattened (UF), x ray beam transmission factor

## Abstract

**Purpose:**

Addition of high Z implants in the treatment vicinity or beam path is unavoidable in certain clinical situation. In this work, we study the properties of radiation interaction parameters such as mass attenuation coefficient (MAC), x ray beam transmission factor (indirect beam attenuation), interface effects like backscatter dose perturbation factor (BSDF) and forward dose perturbation factor (FDPF) for flattened (FF) and unflattened (UF) x ray beams.

**Methods:**

MAC for stainless steel and titanium alloy was measured using CC13 chamber with appropriate buildup in narrow beam geometry. The x ray beam transmission factors were measured for stainless steel and titanium alloy for different field size, off‐axis, and depths. Profile analysis was performed using a radiation field analyzer (RFA) as a function of field size and depth to study the influence of phantom scattering and spectral variation in the beam. In addition, interface effects such as BSDF and FDPF were measured with gafchromic films at maximum BSDF peak position calculated using Acuros XB algorithm and with PPC40 chamber measured at exit side of high Z material, respectively.

**Results:**

The MAC in both cases decreases with increase in energy for stainless steel (SS) and titanium (Ti) alloy. The MAC increases with the change in x ray beam type from flattened to UF beam because of relatively lower mean energy. The x ray beam transmission factor increases with the increase in energy, field size, and depth owing to increase in penetration power phantom scatter, respectively. The measured BSDF and FDPF were found to be in good agreement with AXB algorithm.

**Conclusion:**

The dosimetric properties of x ray photon beam were studied comprehensively in the presence of high Z material like stainless steel and titanium alloy using both flattened and UF beams to understand and incorporate the findings of various parameters in clinical condition due to the variation in energy spectrum from FF to UF x ray beam.

## 1 INTRODUCTION

Flattening filter free or unflattened (UF) x ray beam with higher dose rate is widely used in radiation therapy. It produces 2–4 times high dose rate due to the removal of flattening filter in the beam path which is traditionally used to produce flattened radiation beam.^1^ The unflattened x ray beam considerably decreases treatment time. The head scatter and neutron contaminations are minimized in UF x ray beam delivery owing to lack of flattening filter. In addition, the photon fluence at periphery is minimal compared to that along the central axis of the beam.^2^ The head contamination component (scattered photon, electron, and neutron) of unflattened x ray beam can be incorporated into treatment planning using an accurate dose calculation algorithm. Use of unflattened x ray beams in conformal therapy without beam modulation has been done in small volume tumors up to 3 cm diameter where the flatness of the beam is considered to be same in both the case FF and UF x ray beam delivery. However, the dose rate varies hugely for UF x ray beam. When unflattened beam is used with multi leaf collimator, the advantages of increase in dose rate and beam contamination are slightly compromised.^3^ Mean energy of unflattened x ray beam is relatively lower than flattened beam by means of beam softening (addition of low energy x ray beams) occurs after removing flattening filter from beam path. The difference in mean energy at central axis and the periphery for FF beam is higher, but UF x ray beam possesses little difference in mean energy compared to flattened beam.^4^, ^5^

Unflattened beams are beneficial in treatment techniques like stereotactic radiosurgery (SRS), and hypofractionated stereotactic radiotherapy (SRT), and stereotactic ablative body radiotherapy (SABR), where dose per fraction in the order of 7–24 Gy are delivered. Their characteristic higher dose rates enable fast treatment delivery when compared to using conventional FF beams for a wide range of treatment sites such as brain, head, and neck lymph node boost, lung, abdomen, and prostate. In addition, it is advantageous treating tumors that are prone to organ motion in sites such as lung and abdomen, where the faster dose delivery reduces treatment time thereby increasing efficiency of treatment in managing tumor motion.

Our human body consists of many organs that deviate significantly from unit density like bone, lung, muscle, teeth, air cavities, and small intercalated spaces within bone. In addition to natural in‐homogeneities, artificial bio‐compatible materials such as mandibular reconstruction, hip, leg, and arm prostheses, spinal cord fixation material, surgical rods, stents, and various dental filling may also be present. All these implants are made up of high atomic number (high Z) elements. Materials with an effective atomic number (Z_eff_) greater than that of cortical bone ranging from 6 to 16 (AAPM‐TG65)^6^ are classified as high Z materials. In high energy megavoltage photon beam delivery, these high‐Z materials potentially affect the dose distribution by perturbing the beam. As a consequence, intense dose difference in treatment may result in an inadvertent outcome if their presence in the vicinity of the treatment area is not accounted for accurately by dose calculation algorithms. Increase in population of patients with high‐Z implants undergoing radiotherapy, increases the risk of mistreating a higher percentage of patients. Treating such patients with megavolt x ray beam either by flattened or UF beam may cause a decrease in tumor control due to the reduction in target dose from the shadow of the high‐Z material and increase in future complication rate due to the dose perturbation by the presence of high‐Z material.^7^ However, if dose escalation continues in treating patient, the reduction in tumor dose and increase in dose near bone‐metal or tissue‐metal interface becomes an important factor in compromising tumor control and complications such as high dose to tissue or bone leads to bone necrosis and weakening of the fixation of the implant.

Many users try to avoid using beams through high‐Z material even if it results in additional dose to adjacent critical organs. Some users try to account for the presence of high‐Z material using computerized treatment planning system which uses correction for attenuation of the material. In the recent times, unflattened x ray beam is being used more frequently at treatment sites like prostate with hip prosthesis, spine mets with spinal cord fixation device and less frequently in head and neck treatment with tooth implants.^8^, ^9^, ^10^, ^11^, ^12^, ^13^, ^14^, ^15^, ^16^ The impact of flattened and unflattened beams in the presence of high‐Z implant needs to be studied as both energy types have different energy spectrum, mean energy, and varied fluence. In our study, high‐Z materials of stainless steel and titanium alloy are considered as they are commonly used in implants.^17^, ^18^ The dosimetric characteristics of flattened or UF beam in the presence of high‐Z material have been studied and understood thoroughly.

## 2 MATERIALS AND METHODS

The experiment was carried out in TrueBeam 2.0 (Varian Medical System Inc., Palo Alto, CA, USA) linear accelerator capable of delivering flattened and UF x ray beams of 6 MV (6FF), 10 MV (10FF), 15 MV (15FF) and 6 MV‐FFF (6UF), 10 MV‐FFF (10UF). Even though this study expresses the impact of high‐Z material on flattened and unflattened beams of respective energies, we have included additional flattened 15 MV to examine the impact of high‐Z material. In this study, we used stainless steel (SS316) and titanium alloy (Grade 5) high‐Z materials (Table 1) which are austenitic grades (nonmagnetic). These two high‐Z materials were studied to imitate the biocompatible generally used in implants. The effective atomic number (Z_eff_) of stainless steel (SS316) and titanium alloy (Grade 5) are 29.23 and 22.15 and average mass number (A) of 56.32u, 46.7u, respectively. The composition of stainless steel (SS316) and titanium (Grade 5) material are as follows.^19^ Breadth, width, and thickness of stainless steel and titanium alloy dimensions are 3 × 3×2.5 cm^3^. A special RW3 slab of about 2.5 cm thickness was prepared to accommodate these high‐Z material inserts.

**Table 1:** Physical and chemical properties of stainless steel (SS316) and titanium alloy (Grade 5)

Material	Chemical composition	% of composition	Z	A	ρ (g/cm^3^)
Stainless steel (SS316)	Iron (Fe)	65.35	26	56	7.9
	Chromium (Cr)	17.0	24	52	7.2
	Nickel (Ni)	12.0	28	59	8.9
	Molybdenum (Mo)	2.5	42	96	10.28
	Manganese (Mn)	2.0	25	55	7.43
	Silicon (Si)	1.0	14	28	2.33
	Carbon (C)	0.08	6	12	2.25
	Phosphorus (P)	0.045	15	31	1.83
	Sulfur (S)	0.03	16	32	2.0
Titanium alloy (Grade 5)	Titanium (Ti)	89.55	22	48	4.5
	Aluminum (Al)	6.0	13	27	2.7
	Vanadium (V)	4.0	23	50.9	6.1
	Iron (Fe)	0.25	26	56	7.9
	Oxygen (O)	0.2	8	16	1.43

### 2.A Mass attenuation coefficient

The penetration ability of the beam, that is, mass attenuation coefficient μ/ρ (cm^2^/g) for flattened and unflattened x ray beams for high‐Z material were calculated for narrow beam geometry.^20^ The measurement was carried out in air using CC13 ionization chamber with appropriate buildup to avoid electronic disequilibrium and the chamber was positioned at isocenter and high‐Z material (SS316 and Ti alloy Grade 5) was placed 10 cm above the chamber level exactly equally shadowing around the chamber. A field size of 3 × 3 cm^2^ was opened so that the filed border was inside the high‐Z material. The gap in between chamber and high‐Z material was good enough to avoid any scattering electron reaching chamber to overestimate the result. Measurements were carried out both along the central axis and off‐axis of about 15 cm from central axis longitudinally to quantitate the variation in mean energy at off‐axis that affects mass attenuation coefficient. It was impractical to do measurements at 15 cm along lateral direction because of over traveling of X jaws on either side beyond −2 cm. We assumed that measuring the mass attenuation coefficient at off‐axis can be mirrored on all sides as energy spectrum of all off‐axis is approximately same for flattened and UF x ray beams.

### 2.B Beam transmission and attenuation

The beam transmission and attenuation due to dose perturbation have been measured for both stainless steel (SS316) and titanium alloy (Grade 5) for all available energies of flattened and unflattened x ray beams. The measurement was carried out with CC13 ionization chamber placed at a depth of 10 cm in RW3 slab phantom with 100 cm SSD for 50 MUs. RW3 slab with block of high‐Z materials (SS316 and Ti Grade 5) were inserted at 5 cm depth from the surface. Measurements were made with and without high‐Z material for field sizes 3 × 3 cm^2^ and 30 × 30 cm^2^ at central axis for all available flattened and unflattened x ray beams. The measurement was done at off‐axis distances (3, 5, 8, 10, and 12 cm) along inline and cross‐line direction by keeping constant field size of 30 × 30 cm^2^ with and without high‐Z material at respective off‐axis distances.^21^ The field size of 30 × 30 cm^2^ was used because the maximum filed size is commonly used in clinical circumstances.

### 2.C Profile measurements

The change in dose profile due to high‐Z material for different energies at various depths for given field size was thoroughly studied to quantitate the beam hardening, dose enhancement due to lateral scatter contribution under the shielding for flattened and unflattened beams.

The profile measurements were acquired using a radiation field analyzer (Blue Phantom2 RFA — IBA Dosimetry, Germany) and CC13 field and reference ionization chamber. High Z material was placed at a depth of 5 cm form the water surface using 2 mm perspex tray bridge. Profiles were measured at depths of 10 and 20 cm for field sizes of 5 × 5, 10 × 10, 15 × 15 and 20 × 20 cm^2^ for both flattened and UF x ray beams.

### 2.D Interface effect

The interface effect at the junction of tissue and high‐Z material was studied in an RW3 slab phantom, which has a mass density of 1.043 g/cm^3^ that is approximately equivalent to that of water. Here, two components were studied; the backscattered dose perturbation factor^22^, ^23^, ^24^, ^25^, ^26^, ^27^, ^28^, ^29^, ^30^, ^31^, ^32^, ^33^, ^34^, ^35^, ^36^ at the entrance side of high‐Z inhomogeneity and the forward dose perturbation factor^37^ on the exit side of the inhomogeneity. These two effects were studied for all flattened and unflattened x ray beam energies. Performing measurements at front interface using chamber proved difficult. Hence, we used gafchromic film dosimetry with flatbed scanner (EPSON Expression 10000XL).

The setup for measurement of BSDF was made using small piece of (5 × 5 cm^2^) gafchromic film placed parallel to high Z surface at peak point, calculated by AXB algorithm (±1 mm) in treatment planning system and perpendicular to central axis. Each measurement was done multiple times (5) to reduce the inherent film uncertainty. The high Z materials were (stainless steel and titanium alloy) placed at 5 cm depth with 100 cm SSD. The films were irradiated for 500 MUs each with and without high Z medium. A similar phantom setup was used to determine the FDPF at the exit side of the high‐Z material, where a parallel plate chamber (PPC40) (1 mm thin window and effective point of measurement is just 1 mm below the surface) was used to measure the FDPF.

The reference data for comparing these factors were obtained from Eclipse treatment planning system (TPS) V13.6 (Varian Medical System Inc.) with Acuros XB algorithm, which models the scattering of secondary electrons and photons very well. It also considers mass density of all inhomogeneity rather than electron density. There are several literatures which state that Acuros XB algorithm uses linearized form of Boltzmann transport equation (LBTE) that has equal result compared to Monte Carlo algorithm (MC) with improved calculation time and statistical noise.^38^ The measured values were compared with TPS data, calculated by Acuros AXB algorithm for all flattened and UF x ray beams.

## 3 RESULTS AND DISCUSSION

### 3.A Mass attenuation coefficient

The chamber based measurement of MAC with narrow beam geometry for flattened and unflattened x ray beam shows (Table 2) the differences observed in MAC from central axis to peripheral off‐axis at a distance of 15 cm for stainless steel are 9.6%, 11.6%, 10.6%, 3.0%, and 4.7% and that for titanium alloy are about 8.7%, 11.4%, 10.8%, 3.1%, and 3.2% for 6FF, 10FF, 15FF, 6UF, and 10UF, respectively. The difference in MAC observed at central axis to off‐axis (15 cm) shows that flattened beam has more variation in mean energy than unflattened beam. Introduction of flattening filter in the beam not only reduces dose rate but also mean energy at off‐axis. But for unflattened beam, the difference in mean energy is considerably small from central axis to off‐axis.

**Table 2:** Measured mass attenuation coefficient for stainless steel (SS316) and Titanium alloy (Grade 5) at central and off‐axis

Energy (MV)	Mass attenuation coefficient (μ/ρ cm^2^/g)					
	Stainless steel (SS)			Titanium (Ti) alloy		
	CAX (0 cm)	OAD (15 cm)	% Diff	CAX (0 cm)	OAD (15 cm)	% Diff
6FF	0.04287	0.04700	9.6	0.04541	0.04937	8.7
10FF	0.03536	0.03947	11.6	0.03550	0.03957	11.4
15FF	0.03418	0.03781	10.6	0.03224	0.03575	10.8
6UF	0.04930	0.05079	3.0	0.05055	0.05212	3.1
10UF	0.04010	0.04199	4.7	0.04125	0.04259	3.2

### 3.B Beam transmission and attenuation

The transmission factors measured at 10 cm depth for high Z material such as stainless steel (SS316), placed at 5 cm depth, for 3 × 3 cm^2^ field size the values were 0.6170, 0.6670, 0.6728, 0.5874, and 0.6407 and that for 30 × 30 cm^2^ filed size were 0.6929, 0.7117, 0.7153, 0.6680, and 0.6925 for energies 6FF, 10FF, 15FF, 6UF, and 10UF, respectively. The transmission for titanium (Grade 5) with 3 × 3 cm^2^ field sizes were 0.8008, 0.8330, 08310, 0.7757, and 0.8113 and that for 30 × 30 cm^2^ filed size were 0.8451, 0.8633, 0.8594, 0.8187, and 0.8440 for energies 6FF, 10FF, 15FF, 6UF, and 10UF, respectively.

Differences in transmission factors for stainless steel (SS316) from 3 × 3 cm^2^ to 30 × 30 cm^2^ were 12.3%, 6.7%, 6.3%, 13.7%, and 8.1%; and that for titanium (Grade 5) were 5.5%, 3.6%, 3.4%, 5.5%, and 4.0% for energies 6FF, 10FF, 15FF, 6UF, and 10UF, respectively. The results indicate that transmission factor increases with field size for given energy, and the depth is due to the increase in phantom scattering contribution at measurement point. It suppresses or partially offsets the shielding effect of the high Z (Stainless Steel and Titanium alloy) material. The transmission factor also increases with increase in energy as the raise in penetration power and contribution to increment in transmission due to lateral scattering decreases with increase in energy. For unflattened beam, the difference in transmission factor from 3 × 3 cm^2^ to 30 × 30 cm^2^ was greater compared to flattened beam (e.g., for stainless steel 6FF: 12.3% to 6UF: 13.7% and 10FF: 6.7% to 10UF: 8.1%) because of more beam softening compared to flattened beam.

Furthermore, the transmission factor toward off‐axis (Table 3) in all direction gets marginally decreased due to lower phantom scatter contribution for all energies but the contribution of spectral variation (lower mean energy) at off‐axis with deeper depth is negligible.

**Table 3:** Beam transmission factor of flattened and unflattened x ray beam for stainless steel (SS316) and titanium (Grade 5) at off‐axis

High Z	Off‐axis — Beam transmission factor (30 × 30 cm^2^)										
	Off‐axis distance (OAD) (cm)	6FF		6UF		10FF		10UF		15FF	
		Inline	Cross line	Inline	Cross line	Inline	Cross line	Inline	Cross line	Inline	Cross line
SS	3	0.6985	0.6988	0.6675	0.6731	0.7098	0.7130	0.6978	0.7030	0.7232	0.7225
	5	0.6954	0.6978	0.6669	0.6714	0.7072	0.7122	0.6873	0.6951	0.7191	0.7213
	8	0.6950	0.6910	0.6652	0.6687	0.7065	0.7107	0.6964	0.6943	0.7174	0.7153
	10	0.6839	0.6865	0.6567	0.6652	0.7040	0.7033	0.6953	0.6938	0.7168	0.7147
	12	0.6810	0.6648	0.6635	0.6643	0.7021	0.7012	0.6944	0.6914	0.7167	0.7056
Ti	3	0.8492	0.8363	0.8240	0.8258	0.8561	0.8536	0.8467	0.8501	0.8600	0.8604
	5	0.8426	0.8344	0.8232	0.8228	0.8548	0.8520	0.8443	0.8439	0.8592	0.8591
	8	0.8392	0.8320	0.8191	0.8224	0.8516	0.8468	0.8441	0.8426	0.8576	0.8590
	10	0.8330	0.8248	0.8190	0.8210	0.8510	0.8460	0.8373	0.8382	0.8564	0.8528
	12	0.8293	0.8215	0.8085	0.8173	0.8506	0.8443	0.8370	0.8362	0.8548	0.8509

### 3.C Profile measurement

**Figure 1:**
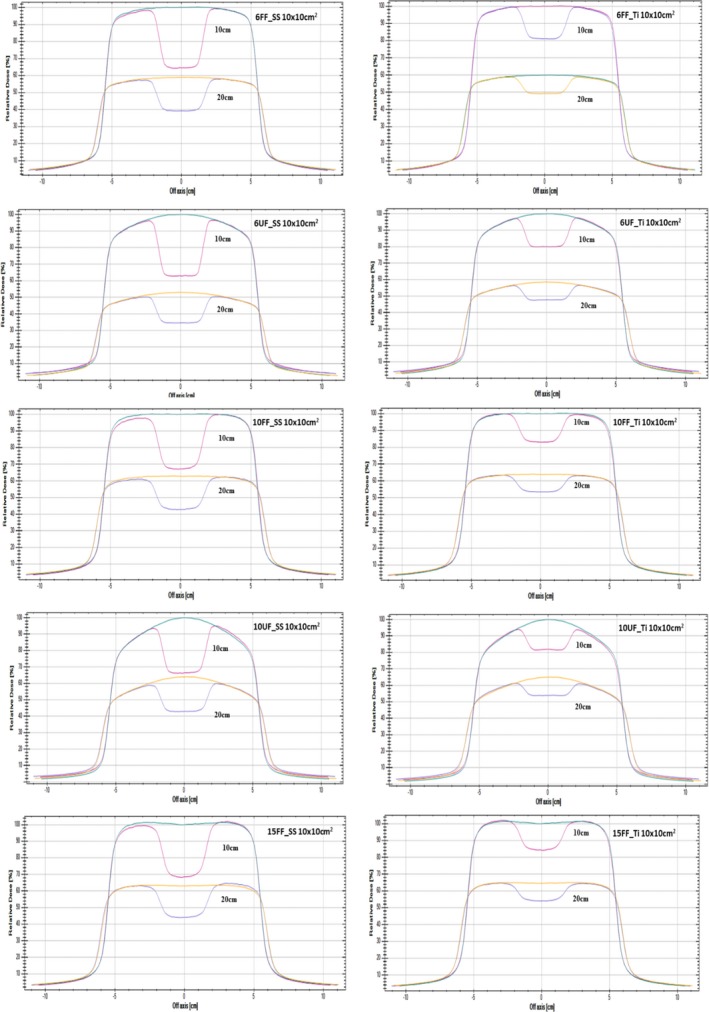
Profile measured of flattened and unflattened x ray beam for stainless steel (SS316) and Titanium (Grade 5) at depth 10 and 20 cm for field size of 10 × 10 cm^2^.

The measured profile under stainless steel and titanium for different energies 6FF, 10FF, 15FF, 6UF, and 10UF at different depths 10 and 20 cm shows (Table 4 and Fig. 1) that the percentage of beam attenuation for flattened and UF x ray beam decreases with increase in depth for all energies. The increase in transmission toward depth shows that increase in phantom scattering contribution also increases from beam softening as it penetrates the high Z material and water medium.

**Table 4:** Beam transmission factor of flattened and unflattened x ray beam for stainless steel (SS316) and titanium (Grade 5) at depth 10 and 20 cm

High Z	Beam transmission factor vs depth (profile measurement)										
	Field size (cm^2^)	6FF		6UF		10FF		10UF		15FF	
		10 cm	20 cm	10 cm	20 cm	10 cm	20 cm	10 cm	20 cm	10 cm	20 cm
Ti	5 × 5	0.7930	0.8590	0.7840	0.8820	0.8340	0.8940	0.8150	0.8756	0.8493	0.8912
	10 × 10	0.8115	0.8930	0.8000	0.8920	0.8310	0.8970	0.8200	0.8895	0.8525	0.8956
	15 × 15	0.8190	0.8990	0.8050	0.8990	0.8350	0.9000	0.8220	0.8927	0.8558	0.9020
	20 × 20	0.8210	0.8994	0.8120	0.9000	0.8360	0.9005	0.8250	0.8951	0.8610	0.9040
SS	5 × 5	0.6250	0.7730	0.6000	0.7767	0.6646	0.7995	0.6640	0.7901	0.6726	0.7804
	10 × 10	0.6450	0.7930	0.6290	0.7805	0.6720	0.8020	0.6660	0.7956	0.6750	0.7911
	15 × 15	0.6590	0.8120	0.6460	0.7832	0.6750	0.8100	0.6680	0.8040	0.6810	0.8086
	20 × 20	0.6670	0.8430	0.6520	0.7914	0.6790	0.8110	0.6760	0.8080	0.6921	0.8136

### 3.D Interface effect

**Figure 2:**
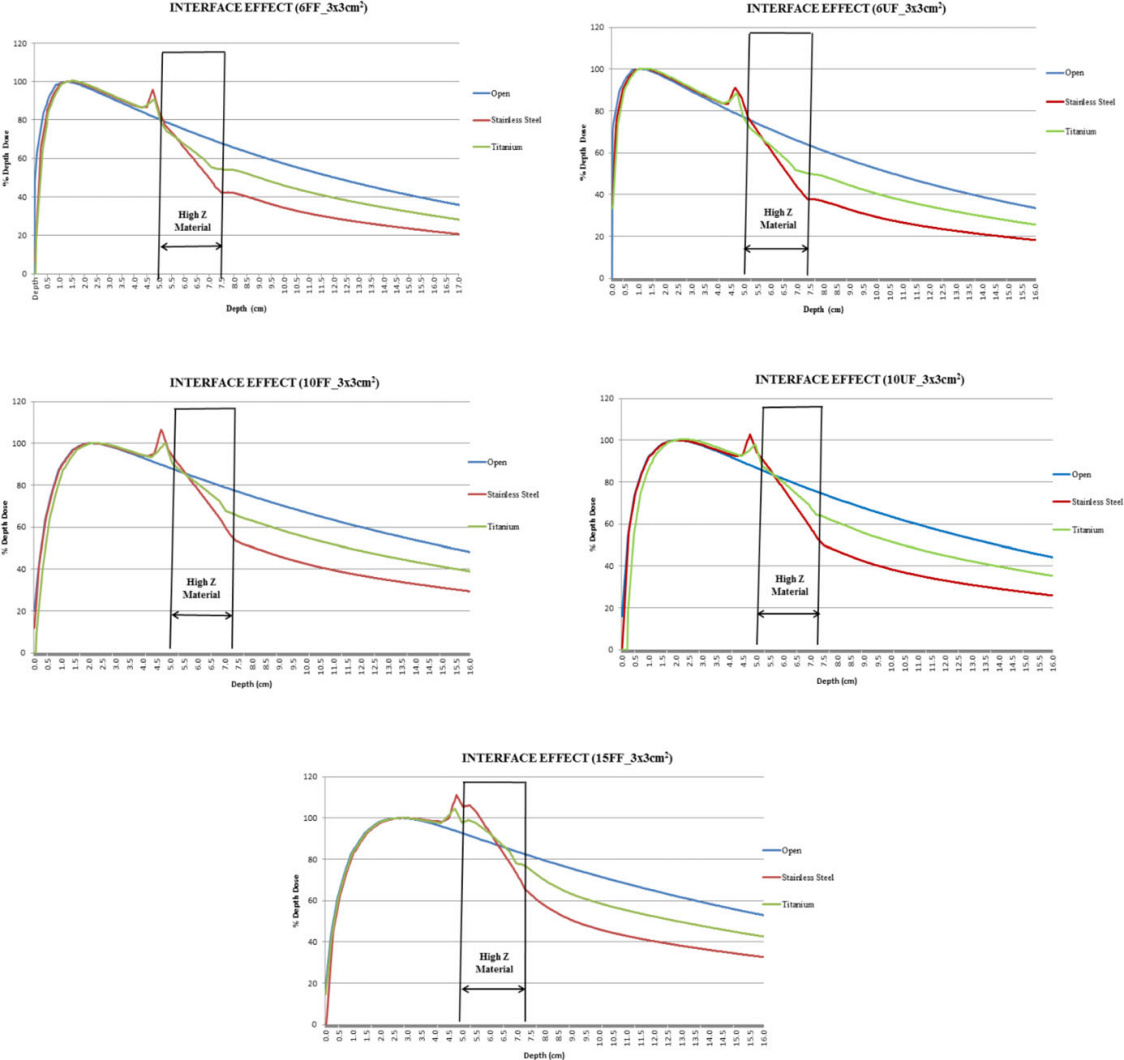
Interface effect observed at high Z material interfaces in PDD calculated by Acuros XB algorithm (Absorbed Dose to Medium) for filed size 3 × 3 cm^2^.

The interface effect like back scatter dose perturbation factor was studied with gafchromic films at peak position (Table 5). The measured values were compared with value obtained from treatment planning system which uses Acuros XB algorithm and the depth of maximum peak of back scatter dose perturbation factor at front junction of RW3‐stainless steel and RW3‐titanium using AXB algorithm for 3 × 3 cm^2^ were 4.7, 4.7, 4.6, 4.6, 4.5 cm and 4.8, 4.8, 4.7, 4.7, 4.6 cm for the energies 6FF, 10FF, 15FF, 6UF, and 10UF for SS316 and Ti‐Grade 5, respectively. For 10 × 10 cm^2^, the depth of maximum BSDF remained the same for SS316 and Ti‐Grade 5 because of lateral scattering contribution due to increase in field size and was found to be 4.8, 4.8, 4.7, 4.7, and 4.6 cm for the energies 6FF, 10FF, 15FF, 6UF, and 10UF, respectively (Fig. 2).

**Table 5:** Calculated and measured BSDF using Acuros XB and film for 3 × 3 cm^2^ and 10 × 10 cm^2^ for flattened and unflattened x ray beams

Energy (MV)	Back scatter dose perturbation factor (BSDF)							
	Stainless steel (SS)				Titanium (Ti) alloy			
	3 × 3 cm^2^		10 × 10 cm^2^		3 × 3 cm^2^		10 × 10 cm^2^	
	Acuros XB	Gafchromic film	Acuros XB	Gafchromic film	Acuros XB	Gafchromic film	Acuros XB	Gafchromic film
6FF	1.132	1.14	1.127	1.13	1.088	1.07	1.086	1.08
6UF	1.113	1.10	1.112	1.08	1.078	1.06	1.105	1.09
10FF	1.163	1.16	1.166	1.15	1.100	1.12	1.106	1.12
10UF	1.147	1.14	1.147	1.14	1.089	1.08	1.099	1.09
15UF	1.177	1.18	1.174	1.18	1.110	1.12	1.112	1.13

**Figure 3:**
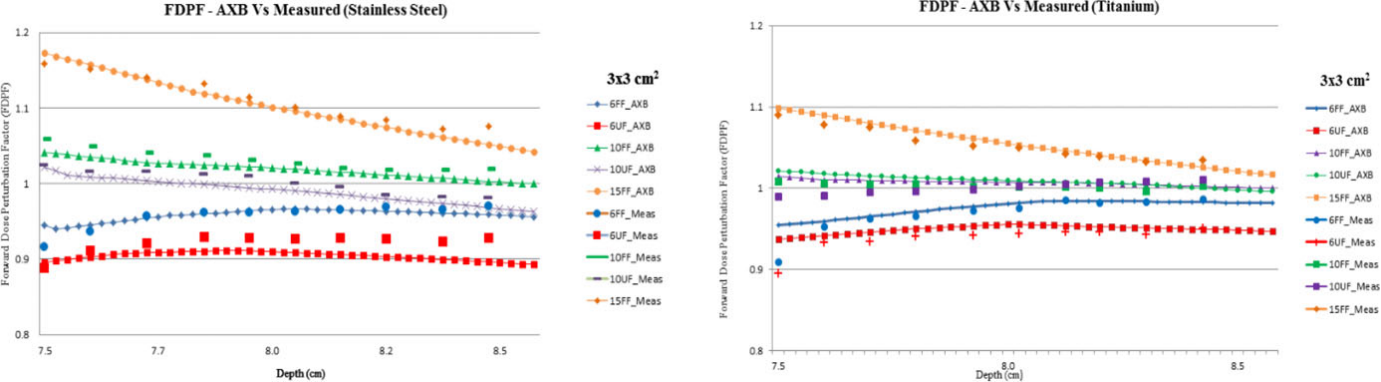
Calculated and measured FDPF using Acuros XB and ionization chamber for flattened and unflattened x ray beams.

The measured BSDF for flattened and unflattened x ray beam was in correlation with the calculated BSDF from Acuros XB algorithm. Figure 3 shows the FDPF for both flattened and unflattened beam measured through AXB algorithm and chamber (PPC40) with field size 3 × 3 cm^2^.

## 4 CONCLUSIONS

The MAC decreases with increase in energy for both flattened and unflattened x ray beam for stainless steel and titanium alloy. MAC is less for unflattened x ray beam compared to flattened x ray beam of same energy since the mean energy for UF x ray beam is lower than flattened beam due to beam softening caused by removal of the flattening filter away from beam path. The measured x ray beam transmission data states that the transmission factor varies with respect to energy (flattened or unflattened), field size and depth. The x ray beam transmission factor increases with the increase in energy because of penetration ability of x ray beam. Furthermore, the x ray beam transmission factor decreases from flattened beam to unflattened beam of same energy since the unflattened x ray beam energy spectrum is softer than flattened beam. The x ray beam transmission increases with the increase in field size for any energy because of lateral contribution of phantom scatter at measurement point. The x ray beam transmission increases with respect to depth, because of lateral scattering contribution, beam softening effect and degradation of attenuation by phantom scattering. On the other hand, the x ray beam transmission factor decreases along off‐axis from central axis of the beam due to gradual reduction in scattering component contribution at measurement point.

The interface effect between RW3 slab and high Z materials of stainless steel and titanium interface was studied in detail using the factors namely BSDF and FDPF. The measured values and that calculated with Acuros AXB algorithm were compared and the result shows that the measured initial buildup of dose in front face of high Z medium characterized by BSDF was found to be in good agreement with Acuros AXB algorithm. Dose buildup due to backscatter electron of unflattened beam is lower than flattened x ray beams due to decrease in backscatter electron. Likewise, the FDPF was measured using parallel plate chamber and was compared with data from Acuros AXB algorithm which shows that the FDPF is low for UF x ray beam than flatted x ray beam due to less forward scatter electron since unflattened x ray beam has less mean energy than flattened x ray beam. The dosimetric properties of x ray photon beam interaction parameters were studied comprehensively in the presence of high Z material like stainless steel and titanium using both flattened and UF x ray beams to understand and incorporate the concept in clinical condition due to the variation in energy spectrum from FF to UF x ray beam in the treatment vicinity with high Z implants.

## CONFLICT OF INTEREST

No conflict of interest.
